# Bispecific antibodies as monotherapy or in combinations for non-hodgkin B-cell lymphoma: latest updates from the American society of hematology 2022 annual meeting

**DOI:** 10.1186/s40164-023-00404-3

**Published:** 2023-04-21

**Authors:** Zhijuan Lin, Long Liu, Zhifeng Li, Bing Xu

**Affiliations:** Department of Hematology, Institute of Hematology, School of Medicine, the First Affiliated Hospital of Xiamen University, Xiamen University, Xiamen, China

**Keywords:** Bispecific antibodies, Follicular lymphoma, Diffuse large B-cell lymphoma

## Abstract

Recent evidence suggests that bispecific antibodies (BsAbs) exhibit promising efficacy and low toxicity even in heavily treated non-Hodgkin B-cell lymphoma (B-NHL). However, the role of BsAbs in previously untreated NHL and the efficacy and safety of BsAbs used in combination remain uncertain. We summarized data published at the 64th American Society of Hematology (ASH) Annual Meeting on BsAb monotherapy or combination therapy for first-line or relapsed/refractory B-NHL. BsAb monotherapy in elderly/unfit newly diagnosed (ND) DLBCL patients achieved ORR and CR rates of 56% and 43%, respectively. In addition, BsAbs combined with chemotherapy or other novel agents raised the ORR higher than 50% without increasing the incidence of grade ≥ 3 CRS. We conclude that BsAb monotherapy or combination therapy for first-line or relapsed/refractory B-NHL has high efficacy and satisfactory safety.

## To the editor

BsAbs can simultaneously bind B-cell lineage-specific surface markers on malignant B cells and CD3 on T cells to activate cellular immunity and induce lysis of malignant B cells. Currently, a variety of CD20×CD3 BsAbs with different structures in the re-engineered Fc domain and the number of Fab arms, including mosunetuzumab, glofitamab, epcoritamab, and odronextamab, are under development as therapies for B-cell lymphomas and may achieve promising response rates in heavily pretreated patients, including those who have progressed after CAR-T therapy. As presented at the 64th American Society of Hematology (ASH) Annual Meeting, several trials are ongoing to further confirm their efficacy and tolerability, as well as optimal timing and combinations. We conducted this review to further elucidate the efficacy and safety of BsAbs for B-NHL.

## BsAb monotherapy

In patients with relapsed/refractory follicular lymphoma (RRFL) who received ≥ 2 prior therapies, mosunetuzumab monotherapy with a maximum of 17 cycles achieved an overall response rate (ORR) and complete response (CR) rate of 77.8% and 60%, respectively [1]. Odronextamab monotherapy until disease progression (PD) or unacceptable toxicity achieved ORR and CR of 81% and 75%, respectively (Table 1) [2]. No grade ≥ 3 cytokine release syndrome (CRS) was observed.

**Table 1:** BsAb monotherapies for B-cell lymphoma

BsAbs	Phase	Settings	N	Median number of prior lines of therapy	Number of treatment cycles	Median follow up (m)	ORR (%)	CR (%)	Median DOR (m)	Median DOCR (m)	Median PFS(m)	CRS Grade ≥ 3 (%)
Mosunetuzumab [1]	II	RRFL	90	3(2–10)	with a CR: Mosun was stopped after 8 cycles; PR or SD: Mosun could be continued for up to 17 cycles	27	77.8	70.0	NR	NR	NR	2.22
Odronextamab [2]	II	RRFL	96	3(2–13)	until PD or unacceptable toxicity	17.3	81	75	18.2	18.2	20.2	0
Odronextamab [3]	II	RR DLBCL	121	2(2–8)	until PD or unacceptable toxicity	17.1	53	37	-	NR	-	0
Epcoritamab [4]	II	RR DLBCL	157	3(2–11)	until PD or unacceptable toxicity	10.7	63	39	12.0	NR	-	2.5%
Mosunetuzumab [5]	I/II	ND DLBCL	53	-	with a CR: Mosun was stopped after 8 cycles; with a PR or SD: Mosun could be continued for up to 17 cycles	23.3	56	43	-	15.8	12 m PFS 39%	0

BsAbs, bispecific antibodies; ORR, overall response rate; CR, complete response; PR, partial response; SD, stable disease; PD, disease progression; DOR, median duration of response; DOCR, median duration of complete response; PFS, progression-free survival; ND, newly diagnosed; RR, relapsed/refractory; FL, follicular lymphoma; DLBCL, diffuse large B-cell lymphoma; NR, not reached; CRS, cytokine release syndrome

In patients with relapsed/refractory (R/R) diffuse large B-cell lymphoma (DLBCL), the ORR and CR of odronextamab were 53% and 37%, respectively [3]. Another BsAb, epcoritamab, also achieved an ORR of 63% and a CR of 39% in RR DLBCL (Table 1) [4]. Grade ≥ 3 CRS was less than 5% in both trials.

Data on mosunetuzumab monotherapy in elderly/unfit newly diagnosed (ND) DLBCL patients have also been reported [5]. Fifty-four patients with a median age of 83 years were enrolled, and the ORR and CR rates were 56% and 43%, respectively. No grade ≥ 3 CRS was observed (Table 1).

In summary, BsAb monotherapy can raise the ORR higher than 50% and the incidence of grade ≥ 3 CRS lower than 5%. Whether the duration of treatment influences the efficacy of BsAbs needs further exploration. The response rate to BsAb monotherapy of aggressive lymphoma was lower than that of indolent lymphoma. Furthermore, the efficacy and safety in elderly/untreated DLBCL patients is worth further exploration.

## BsAbs in combination

In FL, the regimen of epcoritamab in combination with rituximab and lenalidomide (R2) was investigated in both ND and RR settings. The ORR and CR were 95% and 73% in RRFL (n = 56) and 90% and 69% in pervious untreated FL (n = 41) (Table 2) [6, 7]. Neither setting observed severe CRS. Based on these promising data, a phase 3 trial of epcoritamab in combination with R2 versus R2 in patients with RRFL is ongoing (NCT05409066). Meanwhile, mosunetuzumab with lenalidomide as first-line therapy for FL is also under investigation (NCT04792502).

**Table 2:** BsAb combination therapies for B-cell lymphoma

BsAbs	Phase	Settings	N	Median number of prior lines of therapy	number of treatment cycles	Median follow-up (m)	ORR (%)	CR (%)	CRS Grade ≥ 3 (%)
Epcoritamab + R2 [6]	I/II	RRFL	56	1(1–9)	until PD or unacceptable toxicity	4.0	95	73	0
Epcoritamab + R2 [7]	I/II	ND FL	41	-	until PD or unacceptable toxicity	4.4	90	69	0
Epcoritamab + R‑DHAX/C [8]	I/II	RR DLBCL	29 (15 with ASCT)	1	until PD or unacceptable toxicity	9.2	100 (with ASCT) 64 (no ASCT)	80 (with ASCT) 45 (no ASCT)	0
Mosunetuzumab + Pola [9]	Ib/II	RR DLBCL	60	3(1–8)	with a CR: Mosun was stopped after 8 cycles; PR or SD: Mosun could be continued for up to 17 cycles	5.3	72 (age ≥ 65) 54 (age < 65)	56 (age ≥ 65) 38 (age < 65)	0
Glofitamab + RO7227166[10]	I	RR DLBCL	46	3(1–7)	12 cycles		67	39	0
		RR FL	24				91	73	0
Glofitamab + R-CHOP [11]	Ib	ND DLBCL	56	-	8 cycles	5.6	93.5	76.1	0

BsAbs, bispecific antibodies; ORR, overall response rate; CR, complete response; PR, partial response; SD, stable disease; PD, disease progression; ND, newly diagnosed; RR, relapsed/refractory; FL, follicular lymphoma; DLBCL, diffuse large B-cell lymphoma; NR, not reached; R2, rituximab and lenalidomide; ASCT, autologous stem cell transplant; Pola, polatuzumab vedotin; RO7227166, CD19 4-1BBL costimulatory bispecific antibody; CRS, cytokine release syndrome

In patients with RR DLBCL, epcoritamab was investigated in combination with R‑DHAX/C for patients eligible for autologous stem cell transplantation (ASCT) [8]. In 25 patients who proceeded to ASCT, the ORR and CR were 100% and 80%, respectively. Another phase Ib/II trial reported data on mosunetuzumab with the anti-CD79b antibody‒drug conjugate (ADC) polatuzumab vedotin (Pola) [9]. Patients aged ≥ 65 years had an ORR of 72% and a CR of 56%. Additionally, a regimen of glofitamab combined with CD19 4-1BBL (RO7227166) was given in heavily pretreated B-NHL patients [10]. Patients with DLBCL had an ORR of 67% and a CR rate of 70%. None of the above studies revealed high-grade CRS.

Glofitamab was combined with R-CHOP for patients with first-line DLBCL. Fifty-six patients were enrolled; the CR rate was 76.1%, and the ORR was 93.5%. No grade 3–5 CRS events occurred (Table 2). These data demonstrate that glofitamab in combination with other therapies can be effectively and safely used as a first-line treatment for DLBCL.

Although there are no head-to-head trials, BsAbs in combination seem to have a higher response rate and lower incidence of adverse events than BsAb monotherapy.

## Conclusion

Overall, the 64th ASH annual meeting presented a series of efficacy and safety data on BsAbs used in monotherapy or in combination. BsAb monotherapy provides promising efficacy with minimal toxicity and low incidence and severity of CRS. A variety of new trials are ongoing to explore BsAb efficacy and toxicity in different lines of therapy or in combination with other drugs.

## Data Availability

The material supporting the conclusion of this study has been included within the article.

## Abbreviations

BsAbs: bispecific antibodies
ORR: overall response rate
CR: complete response
PR: partial response
SD: stable disease
DOR: median duration of response
DOCR: median duration of complete response
PFS: progression-free survival
ND: newly diagnosed
RR: relapsed/refractory
FL: follicular lymphoma
DLBCL: diffuse large B-cell lymphoma
NR: not reached
CRS: cytokine release syndrome
ADC: antibody‒drug conjugate
